# Hydrops Fetalis Caused by Congenital Syphilis: Case Series and a Comprehensive Review

**DOI:** 10.3390/jcm14113671

**Published:** 2025-05-23

**Authors:** Yuri Yanase, Sirinart Sirilert, Phudit Jatavan, Mallika Pomrop, Krittaya Phirom, Theera Tongsong

**Affiliations:** 1 Department of Obstetrics and Gynecology, Nakornping Hospital, Chiang Mai 50200, Thailand; 2 Department of Obstetrics and Gynecology, Faculty of Medicine, Chiang Mai University, Chiang Mai 50200, Thailand; 3 Department of Pediatrics, Faculty of Medicine, Chiang Mai University, Chiang Mai 50200, Thailand; 4 Department of Obstetrics and Gynecology, Sirindhorn Hospital, Bangkok 10250, Thailand

**Keywords:** anemia, congenital syphilis, fetus, hydrops fetalis, intrauterine transfusion

## Abstract

A total of 30 hydropic fetuses, including 25 cases from published reports and 5 from our own series, were reviewed, validated, and analyzed. This review yielded the following key findings: (1) Unlike most cases of nonimmune hydrops fetalis (NIHF), hydrops caused by syphilis is not only preventable but also treatable, with complete resolution possible when appropriately managed. (2) Syphilis-associated hydrops carries a poor prognosis if timely and appropriate treatment is not administered. (3) Based on limited data, intravenous penicillin G is probably more effective than intramuscular benzathine penicillin in treating hydropic fetuses. (4) Middle cerebral artery peak systolic velocity (MCA-PSV) measurements are increasingly used as a reliable and noninvasive tool for assessing fetal anemia, determining the need for intrauterine transfusion (IUT), and monitoring treatment response. (5) A significant number of cases did not receive prenatal treatment due to false-negative serologic results caused by the prozone effect, as well as the omission of syphilis from the differential diagnosis of NIHF, leading to missed prenatal diagnoses. (6) IUT may help mitigate cellular damage in developing vital organs caused by anemic hypoxia, particularly while awaiting the effects of medical treatment. In conclusion, the modern approach to managing this ancient disease includes: (1) prioritizing intensive intravenous penicillin G therapy over conventional intramuscular benzathine penicillin G; (2) utilizing MCA-PSV in conjunction with other indicators of anemia to monitor its severity; and (3) implementing IUT to prevent anemic hypoxic injury in cases where the hematocrit falls below 30%.

## 1. Introduction

Historically, congenital syphilis was primarily diagnosed postnatally, with most infected neonates exhibiting symptoms by the second month of life [1], while prenatal diagnosis remained relatively uncommon. However, over the past three decades, advancements in high-resolution ultrasound have played an increasing role in the prenatal detection of fetal syphilis. Currently, several affected fetuses can be identified and effectively treated in utero using the standard regimens recommended by the Centers for Disease Control and Prevention (CDC), which are highly effective in both preventing and treating infection in pregnant women [2]. Despite the availability of an effective cure for 80 years, syphilis remains a significant global health concern. Surprisingly, over the past 2 decades, congenital syphilis cases have risen 11-fold in the United States [3]. A similar resurgence has also been documented in several European countries [4,5,6]. The rising stillbirth rate associated with congenital syphilis in the United States has been well documented in recent years, occurring in more than 1 in 20 pregnancies complicated by the infection [7]. To date, severe cases of congenital syphilis, particularly those manifesting as hydrops fetalis secondary to syphilis-associated anemia, continue to be detected in utero and remain associated with high mortality and morbidity, even with standard treatment.

Penicillin remains the standard treatment for all pregnancies complicated by syphilis. However, the specific regimen used for treating syphilis during pregnancy varies across countries and remains a subject of debate [8]. Despite adherence to guideline-based treatment, significant variability has been observed in penicillin levels across maternal serum, cerebrospinal fluid, umbilical cord serum, and amniotic fluid [9]. Cases of fetal treatment failure have been reported, particularly in the presence of sonographic abnormalities such as hydrops fetalis, hepatomegaly, ascites, and placentomegaly [10]. In such cases, the optimal penicillin regimen remains uncertain [11]. The management of hydrops fetalis due to syphilis requires special consideration and may necessitate more effective therapeutic approaches.

To date, despite the availability of prenatal diagnostic tools, there remains limited evidence to guide treatment decisions. Without intervention, hydropic fetuses affected by congenital syphilis would invariably progress to syphilitic stillbirth. Current obstetrical guidelines for the evaluation of non-immune hydrops fetalis (NIHF) provide vague recommendations [12], increasing the risk of missed syphilis diagnoses during NIHF investigations, with significant implications for both individual patient outcomes and public health. Therefore, we conducted this review and case series to compile available cases of syphilis-associated hydrops fetalis treatment reported in the literature and to establish preliminary guidelines for intrauterine management of this serious condition.

## 2. Methods

This descriptive study consists of two parts; a case series and a literature review. This study was ethically approved by the Institutional Review Boards, Faculty of Medicine, Chiang Mai University, Thailand (Research ID: OBG-2567-0207) and the Institutional Review Boards, Nakornping Hospital, Chiang Mai, Thailand (Research ID: NKP No. 092/67).

### 2.1. Literature Review

This review encompasses publications on hydrops fetalis caused by fetal syphilis. The primary objective was to assess treatment approaches and outcomes for syphilis-associated hydrops fetalis. A comprehensive electronic search was conducted using standard databases, including PubMed, Google Scholar, Scopus, and Web of Science, to identify original reports on the prenatal diagnosis of hydrops fetalis due to syphilis, either confirmed prenatally or postnatally, covering reports from 1990 to 2024. The keywords used for the title search were (Hydrops) AND (Syphilis) AND (Pregnancy). Retrieved reports were manually screened based on abstracts or full texts to include only those with detailed prenatal findings and postnatal confirmation of syphilis. For each included report, demographic data, sonographic findings, and obstetric/neonatal outcomes were validated and extracted for analysis. Abnormal ultrasound findings not explicitly mentioned in a report were interpreted as negative. The total cases extracted from the literature were combined with our previously mentioned case series. Reports not describing exclusion of other causes of hydrops fetalis and lacking detailed prenatal ultrasound findings were excluded. For data preparation, all prenatal data and outcomes were extracted and entered as separate variables in the dataset. Methods of treatment and outcomes were examined, validated, and categorized. The frequencies of treatment approaches, responses, and outcomes were analyzed and presented as percentages. The findings based on the literature review and our own case series were combined, validated, and used to propose the preliminary guidelines for intrauterine management of this disorder.

### 2.2. Case Series Presentation

Cases of hydrops fetalis associated with syphilis, collected at Maharaj Nakorn Chiang Mai Hospital and Nakornping Hospital, Chiang Mai, Thailand, between 2005 and 2024, were consecutively included in this study. The inclusion criteria were as follows: (1) pregnancies complicated by hydrops fetalis due to syphilis, confirmed either prenatally or postnatally, and (2) availability of detailed prenatal ultrasound images and video clips evaluated by maternal–fetal medicine (MFM) specialists. All sonographic features, treatment methods, and pregnancy outcomes were reviewed, validated, and analyzed by the authors.

## 3. Results

### 3.1. Review Results

From the search results during the study period (1990–2024) and our experience, a total of 30 cases of hydrops fetalis caused by syphilis were included in the analysis, comprising 25 cases identified in the literature and 5 cases of our own cases, as presented in Table 1. All cases exhibited at least two fluid collections, such as ascites, pleural effusion, pericardial effusion, or subcutaneous edema. Additionally, most cases demonstrated cardiomegaly or an elevated MCA-PSV, suggesting fetal anemia. Hepatomegaly and placentomegaly were also commonly observed.

**Demographic data:** Most patients were adolescents or young adults, with a mean maternal age of 23 ± 5.6 years; however, two-thirds (65.5%) were parous, as presented in Table 2. The majority of reported cases were from Western countries. The gestational age at the first documentation of hydrops fetalis was primarily in the late second or third trimester, with a mean of 28.0 ± 3.6 weeks (range: 19–35 weeks). The mean gestational age at the time of prenatal syphilis diagnosis was 26.7 ± 4.8 weeks. Notably, maternal syphilis was diagnosed postnatally in seven cases (23.3%).

**Treatment:** One-third of cases (33.3%) did not receive prenatal treatment following the diagnosis of non-immune hydrops fetalis (NIHF), possibly due to syphilis not being considered in the differential diagnosis, resulting in a delayed definitive diagnosis until after birth. This finding underscores the need for improved screening and prevention strategies. Notably, five cases exhibited false non-reactive results in prenatal testing due to the prozone effect [13,14]. This finding supports the use of a reverse screening algorithm for syphilis during pregnancy, which can mitigate this issue.

**Standard treatment** of syphilis during pregnancy (2.4 mU BPG weekly, three doses) used in the past tends to change in the most recent years to be more aggressive IV PG 10–14 days, like maternal neurosyphilis, for hydropic fetuses to maximize penicillin levels in fetal blood and tissue. Other medications were not used in any cases. In case of penicillin allergy, desensitization is preferably opted. Note that nine cases (30%) were treated with IV regimen, and more commonly practiced in the more recent years.

**The standard treatment** for syphilis during pregnancy, consisting of BPG 2.4 million units administered weekly for three doses, has shifted in recent years toward a more aggressive intravenous (IV) penicillin G regimen (10–14 days), similar to the treatment for maternal neurosyphilis. This approach is used for hydropic fetuses to maximize penicillin levels in fetal blood and tissue. No alternative medications were reported in any cases. In cases of penicillin allergy, desensitization is the preferred approach. Notably, nine cases (30%) were treated with an IV regimen, a practice that has become more common in recent years.

**MCA-PSV measurement** has been increasingly utilized in recent years as an innovative tool for assessing the severity of fetal anemia, identifying candidates for intrauterine transfusion (IUT), and monitoring treatment response to guide management. More than half of the cases (55.2%) employed MCA-PSV for these purposes.

**IUT** was performed in five cases, with all but one resulting in survival. One fetus died shortly after IUT [23], possibly due to severe preexisting compromise, as indicated by the presence of a sinusoidal fetal heart rate (FHR) pattern prior to the procedure. Other potential contributing factors include volume overload from the transfusion or placental abruption secondary to cordocentesis. Additionally, this case received an intramuscular rather than an intravenous penicillin regimen. While IUT has played an increasingly important role in recent years, careful monitoring for volume overload remains essential.

Syphilis-associated hydrops fetalis could be reversible in utero with appropriate medical treatment, distinguishing it from most cases of NIHF caused by other etiologies. In response to treatment, nearly 80% of cases demonstrated either partial improvement (39.1%); characterized by a reduction in severity of hydrops or complete resolution (39.1%), defined as the intrauterine disappearance of hydrops. Notably, the IV penicillin regimen appeared more effective than the IM regimen, although the sample size was too small for a definitive comparison. Additionally, IUT seemed beneficial during the early phase of treatment, before the full therapeutic effect of medication was achieved; however, further studies are needed to confirm this observation.

**Pregnancy Outcomes:** The mean gestational age at birth was 32.4 ± 4.8 weeks. The preterm birth rate (<37 weeks) was 71.4%, and the cesarean section rate was 52%, with the majority performed due to fetal distress (non-reassuring fetal status), accounting for more than half of the cases. The perinatal mortality rate was 33.3%, comprising intrauterine fetal demise (20.0%) and neonatal death (13.3%).

Among the 10 cases with perinatal mortality, five had not received prenatal treatment, while four had been treated with intramuscular benzathine penicillin G (IM BPG), and one had received intravenous penicillin G (IV PG) without undergoing intrauterine transfusion (IUT). The final case involved severe hydrops fetalis, leading to termination one week after the initiation of treatment [27]. All surviving newborns were treated for congenital syphilis, even in cases where no residual signs of the disease were evident.

### 3.2. Case Series Presentation

**Case 1 (2017)**

A 22-year-old woman, G1P0, residing in Chiang Mai, presented for her first antenatal care visit at 26 weeks of gestation. Her medical and family histories were unremarkable, with no history of sexually transmitted infections or genital ulcers. The pregnancy had been uneventful up to that point. Routine antenatal laboratory investigations were within normal limits, except for a positive Venereal Disease Research Laboratory (VDRL) test at a titer of 1:32, which was confirmed by a positive Treponema pallidum hemagglutination assay (TPHA). Her hemoglobin level was 11 mg/dL, indicating a normal blood count. Based on these findings, she was diagnosed with late latent syphilis. Physical examination revealed normal vital signs and systemic findings. The uterine size was consistent with gestational age, and fetal heart rate was normal. Ultrasound evaluation demonstrated normal fetal biometry corresponding to 26 weeks of gestation. However, the fetus exhibited hepatomegaly and cardiomegaly, with a cardio-thoracic ratio (CTR) of 0.63 and holo-systolic tricuspid regurgitation. The middle cerebral artery peak systolic velocity (MCA-PSV) was 73.11 cm/s (2.17 MoM). Features of hydrops fetalis, including ascites, pericardial effusion, and subcutaneous edema, were also observed. The patient received the first dose of intramuscular benzathine penicillin G (2.4 million units), with a planned weekly regimen for three weeks. Despite treatment, hydrops fetalis persisted at 30 weeks of gestation. Antepartum surveillance revealed non-reassuring fetal status, including a non-reactive non-stress test (NST) and reverse end-diastolic velocity in the umbilical arteries. Due to signs of fetal distress, an emergency cesarean section was performed, delivering a hydropic female neonate, weighing 1328 g, with Apgar scores of 2 and 3T at 1 and 5 min, respectively. The newborn was diagnosed with congenital syphilis complicated by hydrops fetalis and anemia. Despite resuscitative efforts, the neonate succumbed three hours after birth. Postnatal investigations confirmed the diagnosis of congenital syphilis.

**Case 2 (2018)**

A 23-year-old woman, G3P2, attended her first antenatal clinic visit at 30 weeks of gestation. She was residing in Chiang Mai. Her medical and family histories were unremarkable. She denied any history of sexually transmitted infections or genital ulcers. Notably, she had not received any prenatal care during the current pregnancy. Her two previous pregnancies were uneventful, resulting in term vaginal deliveries of healthy infants. The current pregnancy had also been uneventful. Upon physical examination, her vital signs and systemic findings were normal. Obstetric examination was unremarkable. The uterine size was consistent with gestational age, with a fundal height of 30 cm, and the fetus was in a vertex presentation with a normal heart rate. Routine antenatal laboratory tests were within normal limits, except for a positive Venereal Disease Research Laboratory (VDRL) test at a titer of 1:32. A Treponema pallidum hemagglutination assay (TPHA) was performed, confirming the diagnosis of syphilis. Fetal ultrasound examination demonstrated normal biometry corresponding to 30 weeks of gestation. However, hepatomegaly, placentomegaly (placental thickness of 4.2 cm), and global cardiomegaly were observed, with a cardio-thoracic ratio (CTR) of 0.66 and trivial tricuspid regurgitation. The middle cerebral artery peak systolic velocity (MCA-PSV) was 81.3 cm/s (2.01 MoM). Hydrops fetalis was evident, characterized by ascites, pleural effusion, pericardial effusion, and subcutaneous edema. The patient was treated with intravenous penicillin G (5 million units every 4 h for 14 days). Fetal blood sampling and intrauterine transfusion were offered; however, the patient opted for close monitoring of MCA-PSV and fetal hydrops rather than undergoing cordocentesis. Follow-up ultrasound demonstrated improvement in MCA-PSV, which returned to the normal range within one week of treatment. All signs of hydrops fetalis had completely resolved by two weeks after the initial treatment. The remainder of the pregnancy was uneventful. At 38 weeks of gestation, the patient underwent spontaneous labor and vaginal delivery, giving birth to a healthy female newborn weighing 2420 g, with Apgar scores of 9 and 10 at 1 and 5 min, respectively. The neonate’s VDRL test was positive at a titer of 1:4, but cerebrospinal fluid (CSF) VDRL was non-reactive. The newborn was treated with aqueous penicillin G (50,000 U/kg/day intravenously every 8 h). Additional investigations, including TORCH screening, parvovirus B19 testing, karyotyping, and hematologic studies, were performed to evaluate other potential causes of hydrops fetalis; all results were within normal limits. The newborn remained healthy and was discharged home on day 7 of life.

**Case 3 (2008)**

A 19-year-old woman, G1P0, residing in Chiang Mai, presented for her first antenatal care visit at approximately six months of gestation, although the exact date was uncertain. Her medical and family histories were unremarkable, with no history of sexually transmitted infections or genital ulcers. The current pregnancy was unplanned but had been uneventful. Physical examination findings were unremarkable. The uterine size was 4 cm above the umbilicus, and the fetal heart rate was normal. Routine antenatal laboratory investigations were within normal limits, except for a positive Venereal Disease Research Laboratory (VDRL) test at a titer of 1:1024, which was confirmed by a positive Treponema pallidum hemagglutination assay (TPHA). Her hemoglobin level was 12 mg/dL. Fetal ultrasound examination revealed biometry corresponding to 24 weeks of gestation. However, the fetus exhibited hepatosplenomegaly and cardiomegaly, with a cardiothoracic ratio (CTR) of 0.60 in the absence of structural abnormalities. The middle cerebral artery peak systolic velocity (MCA-PSV) was 70.4 cm/s (2.23 MoM). Hydropic features were present, including marked ascites, pericardial effusion, and pleural effusion. Additionally, an enlarged placenta (measuring 6.8 cm in thickness) and polyhydramnios were noted. The patient received the first dose of intramuscular benzathine penicillin G (2.4 million units), with a planned weekly regimen for three weeks. Despite treatment, hydrops fetalis persisted at 25 weeks of gestation. Spontaneous labor occurred shortly after she received the second dose of benzathine penicillin, leading to a normal vaginal delivery of a male stillborn hydropic fetus weighing 850 g. The hydropic placenta weighed 540 g, and postnatal pathological examination confirmed the diagnosis of congenital syphilis. The patient and her husband completed the full course of penicillin treatment during postpartum follow-up.

**Case 4 (2009)**

A 28-year-old woman, G3P1, attended her first antenatal clinic visit at 25 weeks of gestation. She was residing in Chiang Mai. Her medical and family histories were unremarkable. Her first pregnancy was uneventful, resulting in a normal vaginal delivery of a healthy newborn. However, her second pregnancy ended in miscarriage, and she had not attended antenatal care. She had no history of sexually transmitted infections or genital ulcers. Notably, she had not received any prenatal care during the current pregnancy; however, the pregnancy had been uneventful. Upon physical examination, her vital signs and systemic findings were normal. The uterine size was consistent with gestational age, with a fundal height of 26 cm and a normal fetal heart rate. Routine antenatal laboratory tests were within normal limits, except for a positive Venereal Disease Research Laboratory (VDRL) test at a titer of 1:1024. A Treponema pallidum hemagglutination assay (TPHA) was performed, confirming the diagnosis of syphilis. Fetal ultrasound demonstrated biparietal diameter and femur length corresponding to 25 weeks of gestation, while the abdominal circumference was consistent with 28 weeks. Hepatomegaly, placentomegaly (placental thickness of 4.5 cm), and global cardiomegaly were observed, with a cardiothoracic ratio (CTR) of 0.68. Hydrops fetalis was evident, characterized by ascites, pleural effusion, pericardial effusion, and subcutaneous edema. The middle cerebral artery peak systolic velocity (MCA-PSV) was 76.3 cm/s (2.35 MoM). Additional workup was negative for TORCH screening, parvovirus B19 testing, and fetomaternal hemorrhage. The patient was treated with intravenous penicillin G (5 million units every 4 h for 14 days). Cordocentesis revealed a fetal hematocrit of 7.2 g/dL. Intrauterine transfusion was successfully performed, though transient fetal bradycardia was noted shortly after the procedure. Follow-up ultrasound demonstrated improvement in MCA-PSV, which returned to the normal range within 24 h of treatment. All signs of hydrops fetalis had completely resolved by two weeks after the initial treatment. The remainder of the pregnancy was uneventful. At 38 weeks of gestation, the patient underwent spontaneous labor and vaginal delivery, giving birth to a healthy male newborn weighing 3200 g, with Apgar scores of 5 and 9 at 1 and 5 min, respectively. The neonate’s VDRL test was positive at a titer of 1:8, but cerebrospinal fluid (CSF) VDRL was nonreactive. The newborn was treated with aqueous penicillin G (50,000 U/kg/day intravenously every 8 h). The infant remained healthy and was discharged home on day 8 of life.

**Case 5 (2024)**

A 22-year-old woman, G3P2, residing in Bangkok, presented for her first antenatal care visit at 26 weeks of gestation. Her medical and family histories were unremarkable, with no history of sexually transmitted infections or genital ulcers. Her two previous pregnancies were uneventful, resulting in vaginal deliveries of healthy newborns. She had not attended antenatal care during the current pregnancy. Physical examination findings were unremarkable. The uterine size was appropriate for gestational age, and the fetal heart rate was normal. Routine antenatal laboratory investigations were within normal limits, except for a positive Venereal Disease Research Laboratory (VDRL) test at a titer of 1:128, which was confirmed by a positive Treponema pallidum hemagglutination assay (TPHA).

**Figure 1:**
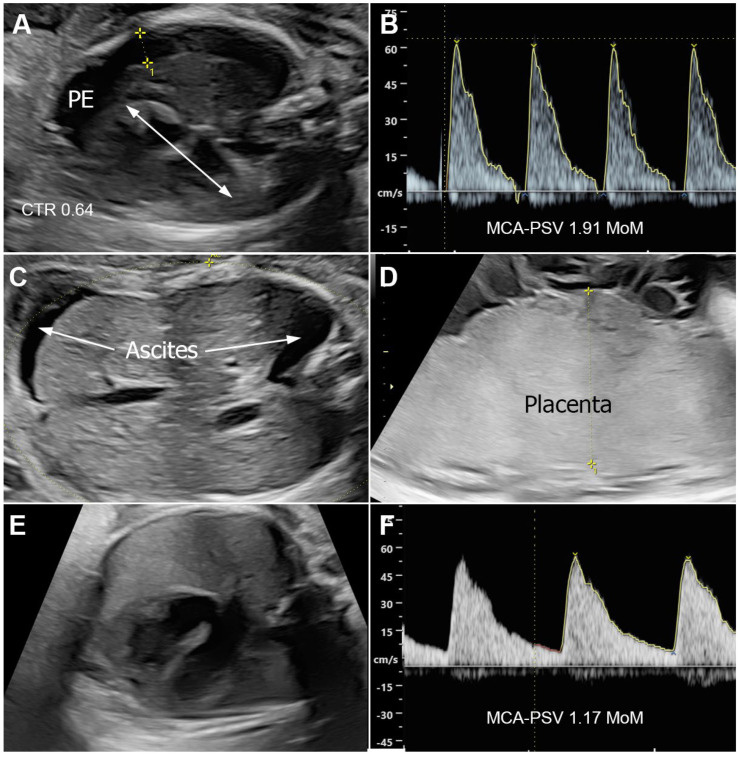
Ultrasound findings of Case 6; (**A**–**D**) (26 weeks) show hydrops; pleural effusion (PE) and cardiomegaly (**A**), high MCA–PSV (**B**), ascites (**C**), and placentomegaly (**D**); (**E**,**F**) (33 weeks) show disappearance of hydrops; no pleural effusion (**E**) and normal MCA–PSV (**F**).

Fetal ultrasound examination revealed biometry corresponding to 26 weeks of gestation. However, the fetus exhibited ascites, right-sided pleural effusion, and subcutaneous edema, leading to a diagnosis of hydrops fetalis (Figure 1A–D). There was evidence of placentomegaly or cardiomegaly, no structural abnormalities, and no tricuspid regurgitation. The middle cerebral artery peak systolic velocity (MCA-PSV) was 64.8 cm/s (1.91 MoM). Doppler studies of the umbilical artery, umbilical vein, and ductus venosus were normal. No other structural anomalies were observed. Other potential causes of hydrops fetalis were not identified, and a provisional diagnosis of hydrops fetalis secondary to syphilis was made. Cordocentesis for fetal anemia evaluation and potential intrauterine transfusion was offered. However, the patient and her husband opted for close monitoring with serial MCA-PSV assessments rather than an invasive procedure. The patient received intramuscular benzathine penicillin G (2.4 million units) once weekly for three weeks. A follow-up ultrasound four days later showed no significant change in hydropic signs or MCA-PSV. By 28 weeks of gestation, ascites and pleural effusion had subjectively improved. By 33 weeks, all hydropic signs had resolved and were not observed in subsequent biweekly follow-up ultrasounds and MCA-PSV had decreased to 54.5 cm/s (1.31 MoM) (Figure 1E,F). Non-stress tests remained reactive throughout the third trimester. At 38 weeks of gestation, the patient experienced spontaneous labor and underwent vaginal delivery, giving birth to a healthy female newborn weighing 2850 g, with Apgar scores of 8 and 10 at 1 and 5 min, respectively. All neonatal workup results were within normal limits, with no signs of congenital syphilis. The newborn was treated with aqueous crystalline penicillin G at a dose of 143,000 units intravenously every 12 h from days 1 to 7, followed by 143,000 units intravenously every 8 h from days 8 to 10 (50,000 units/kg/dose). The infant remained healthy, with normal findings at the 2-month follow-up.

## 4. Discussion

The insights gained from this case series and review are as follows: (1) Unlike most cases of NIHF, hydrops fetalis caused by syphilis is not only preventable, as is well established, but also treatable, with complete resolution possible when appropriately managed. (2) Syphilis-associated hydrops is associated with a very poor prognosis if proper treatment is not administered. (3) Even when mothers have early or latent syphilis, intensive treatment using a neurosyphilis regimen (intravenous penicillin G) is likely to be more effective for hydropic fetuses. (4) MCA-PSV measurements are increasingly used as an effective and non-invasive tool for assessing fetal anemia, determining the need for IUT, and monitoring treatment response. (5) A significant number of cases fail to receive prenatal treatment due to false-negative serologic results caused by the prozone effect, as well as the omission of syphilis from the differential diagnosis of NIHF, leading to missed prenatal diagnoses. (6) IUT is likely beneficial in mitigating cellular damage in developing vital organs caused by anemic hypoxia, particularly while awaiting the effects of medical treatment.

### 4.1. Intramuscular BPG vs. Intravenous PG Regimen

NIHF represents the most severe manifestation of fetal syphilis, occurring in utero. Conventional treatment guidelines recommend intramuscular benzathine penicillin G (IM BPG) administered weekly for three doses, which is generally sufficient for preventing fetal transmission in cases of uncomplicated maternal syphilis. However, in the presence of hydrops fetalis, this regimen is associated with a high failure rate and significant morbidity and mortality. Therefore, hydrops fetalis should be considered a special circumstance requiring alternative management strategies. Based on the limited evidence available in this review, NIHF appears to necessitate a more aggressive treatment approach, similar to that used for maternal neurosyphilis, even when the mother has late latent syphilis without clinical manifestations. The rationale for this approach is to maximize therapeutic effects within a short time frame, given that the fetus is already severely anemic. Without timely intervention, cellular damage secondary to anemic hypoxia in developing vital organs may progress beyond the point of compensation, ultimately leading to irreversible deterioration. The standard IM BPG regimen may require more time to exert its full therapeutic effect, potentially allowing ongoing tissue damage due to worsening anemic hypoxia.

This concern is exemplified by a case reported by Galan et al. [16], in which a fetus with NIHF secondary to syphilis was initially treated with intramuscular BPG. Despite treatment, hydropic signs worsened 10 days after initiation. However, following a switch to intravenous penicillin G (IV PG), the fetus demonstrated complete resolution of hydrops within 10 days. Similarly, Chen et al. [20] successfully treated a fetus with NIHF secondary to syphilis using a 14-day IV PG regimen in conjunction with IUT, resulting in complete resolution of hydrops within two weeks. Our own experience with Case III yielded similar success.

Despite these findings, the number of reported cases remains limited, and some cases have shown complete resolution of hydrops fetalis with intramuscular BPG alone. Therefore, while IV PG appears to be superior to IM BPG in cases where hydrops fetalis has already developed, further comparative studies are urgently needed to establish the optimal treatment regimen.

### 4.2. The Role of Intrauterine Blood Transfusion (IUT)

The role of IUT in the management of anemic hydrops secondary to syphilis remains unclear. Some experts, such as Rac et al. [1,2], do not recommend IUT, as adequate treatment with BPG is believed to reverse the hematologic abnormalities associated with fetal syphilis [1,29]. However, their series did not include severe cases of hydrops fetalis, as presented in this review. Notably, this review demonstrates that several cases showed no response or only a partial response, without complete resolution of hydrops fetalis, despite receiving the standard intramuscular BPG regimen or even an intravenous penicillin G (PG) regimen. This finding suggests the necessity of additional therapeutic modalities. It is well established that fetal anemic hypoxia can lead to cellular damage in developing vital organs such as the heart and brain [30,31]. Therefore, we believe that, in cases where the hydropic fetus has already been compromised due to anemic hypoxia, IUT is both necessary and urgent to improve tissue oxygen perfusion before cellular destruction in vital organs progresses beyond the point of complete recovery with medication alone. Theoretically, IUT enhances oxygen delivery to tissues and augments the effects of pharmacologic treatment in peripheral organs. Accordingly, this review suggests that IUT is warranted in select cases confirmed to have severe anemia. While no specific guidelines exist for IUT in syphilis-associated hydrops fetalis, the approach recommended by the Society for Maternal–Fetal Medicine (SMFM) [32] and the American College of Obstetricians and Gynecologists (ACOG) [33] for fetal anemia secondary to Rh isoimmunization or parvovirus B19 may reasonably be applied in cases of syphilis-associated anemia. In this strategy, non-invasive measurement of the MCA-PSV is used to identify fetuses at high risk for anemia, guiding the decision for cordocentesis to assess fetal hematocrit. IUT is recommended if the hematocrit is below 30%. Based on the findings of this review, IUT is typically required only once at the critical stage of anemic hypoxia, with no further need once the pharmacologic treatment takes full effect.

**Additional noteworthy observations** include the following: (1) Unlike NIHF caused by other etiologies, which is typically irreversible, hydrops secondary to syphilis is reversible and can completely resolve with appropriate treatment [16,20,26,28]. However, without intervention, the prognosis is extremely poor [13,18,19,25]. In this review, one-third of cases presenting with hydrops-associated fetal distress resulted in stillbirth or neonatal death. (2) Syphilis should always be considered in the differential diagnosis of NIHF. Several cases of NIHF were postnatally confirmed to be caused by syphilis after birth or stillbirth, having failed to receive appropriate prenatal treatment. Moreover, some cases were diagnosed prenatally at an advanced stage, when the likelihood of therapeutic response was low, especially in fetuses exhibiting distress, such as a sinusoidal pattern, prior to treatment initiation [23]. (3) The sonographic features of NIHF due to syphilis are consistent with those of other anemia-related conditions, including elevated MCA-PSV to varying degrees, cardiomegaly, hepatomegaly, and placentomegaly. Notably, all cases included in this review involved mothers who had never received treatment for syphilis before the diagnosis of hydrops. No cases occurred during or after adequate treatment, indirectly suggesting that early maternal treatment could effectively prevent NIHF. Accordingly, this review illustrates the importance of syphilis screening and early treatment to prevent severe complications. (4) A significant number of cases were affected by the prozone effect, leading to false-negative results and subsequent lack of prenatal treatment [13,18]. These findings highlight the advantages of the reverse screening algorithm, which can help detect such cases more effectively. (5) Other major adverse pregnancy outcomes were relatively high among those pregnancies, such as the increased rates of preterm birth, cesarean section, and fetal distress.

**Clinical implication:** This review highlights hydrops fetalis due to fetal syphilis, a condition associated with a high mortality rate, despite being preventable through effective screening and early treatment. Many severe cases result from inadequate screening or false non-reactive serologic results due to the prozone effect. Therefore, the implementation of a reverse screening algorithm should be strongly considered in routine clinical practice. Clinically, this review urges obstetrical care providers to enhance syphilis screening and to maintain a high index of suspicion for syphilis when evaluating severe fetal anemia. Syphilis should always be included in the differential diagnosis of NIHF. While the optimal management approach remains uncertain, a combination of intravenous penicillin G (IV PG), middle cerebral artery peak systolic velocity (MCA-PSV) measurement, and selective IUT in cases of severe anemia should be considered.

**Research implication:** This review highlights the scarcity of the current literature to guide the prenatal management of hydropic fetuses secondary to syphilis. Information sharing will be essential in building a modern knowledge base for the treatment of this resurging infection. We encourage obstetricians and perinatologists to report their experiences with various management approaches to contribute to the accumulation of cases in the literature, facilitating the future development of clinical guidelines. Additionally, studies or larger case series evaluating the effectiveness of intensive intravenous penicillin G (IV PG) regimens and IUT, with a high degree of methodological homogeneity, are urgently needed.

## 5. Conclusions

This review highlights a paradigm shift in the management of this ancient disease, moving from the conventional intramuscular BPG regimen to intravenous penicillin G, combined with selective IUT under the guidance of modern MCA-PSV assessment. This approach has resulted in notable improvements in perinatal outcomes. However, this conclusion is based on a limited number of cases, and further accumulation of clinical data on the treatment of hydrops fetalis due to syphilis is urgently needed.

## Figures and Tables

**Table 1:** Details of each reported case included in the review.

No.	Author (Year-Country)	Age	Parity	GA at Diagnosis of NIHF	GA at Diagnosis of Syphilis	MCA-PSV	MoM	Hb	Treatment	IUT	Response	GA at Birth	Route	BW	Sex	Neonatal Outcomes	Note
1	Bercowitz (1990-USA) [13]	26	G8P3	32	Postnatal	no			no	no		32	CS	2525	M	Survive	Non-reactive (prozone); CS
2	Bercowitz (1990-USA) [13]	21	G3P1	32	Postnatal	no			no	no		32	CS	1460	M	Survive	Non-reactive (prozone); CS
3	Bercowitz (1990-USA) [13]	36	G6P4	Term	Postnatal	no			no	no		Term	CS		M	ND	Non-reactive (prozone); CS (petechiae, hepatosplenogemagly, thrombocytopenia), died d1
4	Bercowitz (1990-USA) [13]	30	G4P1	27	27	no			no	no				2070		DFU	Non-reactive (prozone); CS (HD, petechiae, splenogemagly, thrombocytopenia)
5	Barton (1992-USA) [14]	21	G2P1	31	31	no			IM	no	partial	31	CS	3540	F	Survive	CS (metaphyseal rarefaction in all long bones)
6	Barton (1992-USA) [14]	18	G2P0	35	35	no			NS	no	partial	35	CS	2700	F	Survive	Fetal distress, HF present
7	Barton (1992-USA) [14]	21	G2P0	34	34	no			IM	no	partial	34	CS	2530	M	Survive	Fetal distress
8	Hallak (1992-USA) [15]	16	G2P1	28	28	no			IV	no	partial	28	CS	1546	F	Survive	Fetal distress, HF present (improved), ascites neurosyphilis
9	Galan (1993-USA) [16]	21	G2P1	24	24	no			IV	no	complete	37	Vg			Survive	IM BPG 1 dose HD worsening → change to IV PG complete disappeared in 3 weeks
10	EITabbakh (1994-USA) [17]	22	G4P2	26	26	no			IM	no	complete	38	Vg	2310	M	Survive	Allergic to penicillin → desensitization; HD disappeared, no CS
11	Levine (1998-USA) [18]	31	G3P2	29	Postnatal	no			no	no		29	CS	1825	M	ND	Non-reactive (prozone); CS Hydrops not improved, RPR 1:1024
12	TANER (2004-Turkey) [19]	20	G1P0	28	Postnatal	no			no	no	no response	28		2200	F	DFU	DFU (1 d after diagnosis)
13	Chen (2010-Canada) [20]	17	G1P0	27	28	yes	1.55	5.5	IV	yes	complete	35	Vg	2390	M	Survive	Penicillin 4 mU IV 14 d; HD disappeared (2 wk of IUT 30 wk), no CS
14	Arujo (2012-Brazil) [21]	21	G1P0	25	32	no			IM	no	partial	33	CS	3095		Survive	PGS Gradually improved
15	Mace (2014-France) [22]	17	G1P0	34	Postnatal	yes	1.5	8.4	no	yes	partial	35	CS	2300	F	Survive	Not obvious improved, titer 1/5120, Start Extencillin D3 after birth
16	Mace (2014-France) [22]	27	NS	26	17	yes	2		no	no		26	Vg	720	F	DFU	
17	Fuchs (2016-Canada) [23]	19	G2P0	23	23	yes	2.1	8.2	IM	yes	no response	23	Vg	858	F	DFU	Fetal distress; sinusoidal FHR prior to IUT; MCA decreased to 0.81 after BPG/IUT
18	Duby (2019-Canada) [24]	28	G5P2	28	31	yes	1.84	5.5	no	yes	partial	31		1710		Survive	At birth, hydrops was present; NB improve with IV PG 14 d
19	Ramis (2019-Spain) [25]	23	G3P0	32	Postnatal				no	no		32	CS	2510	M	ND	Fetal distress; no treatment; die d2 after birth
20	Camacho-Montano (2021-Colombia) [26]	17	G2P1	26	26	yes	mild anemia		IV	no	partial	37	Vg	2820	M	Survive	HD improved in 14 d; negative for congenital syphilis
21	Camacho-Montano (2021-Colombia) [26]	28	G2P1	28	28	yes	no anemia		IV	no	complete	37	Vg	2325	M	Survive	MCA-PSV normal; HF disappeared
22	Camacho-Montano (2021-Colombia) [26]	20	G2P1	30	16	yes	no anemia		IM	no	partial	30	CS	1260	F	Survive	Fetal distress; improve after birth
23	Camacho-Montano (2021-Colombia) [26]	18	G3P2	29	29	yes	severe anemia		IV	no	complete	36	Vg	2460		Survive	HD resolve in 7 d
24	Rosenthal (2022-Canada) [27]	29	G4P3	19	19	yes	1.37		IV	no	no response	21	Vg	747	F	DFU	Primary Sy, 9 d worsening HD (99th centile); placenta 387 (twice normal)
25	Dinicu (2023-USA) [28]	38	G5P3	28	29	yes	1.49		IM	no	complete	37	CS		M	Survive	BPG IM, 2.4 mU (3 doses) Hydrops disappeared in 6 days after penicillin first dose; pre-eclampsia with severe features
26	This study (2025-Thailand)	22	G1P0	26	26	yes	2.17		IM	no	no response	30	CS	1328	F	ND	Fetal distress; BPG IM, 2.4 mU (2 dose); HF worsening, died 3 h after birth
27	This study (2025-Thailand)	23	G3P2	30	30	yes	2.01		IV	no	complete	38	Vg	2420	F	Survive	HF disappeared in 3 weeks after penicillin first dose (no JHR)
28	This study (2025-Thailand)	19	G1P0	24	24	yes	2.23		IM	no	no response	25	Vg	850	M	DFU	BPG IM, 2.4 mU (1 dose); Hydrops fetalis, worsening
29	This study (2025-Thailand)	19	G1P0	24	25	yes	2.35	7.2	IV	yes	complete	38	Vg	3200	M	Survive	Hydrops disappeared in 3 weeks after IUT
30	This study (2025-Thailand)	22	G3P2	26	26	yes	1.91		IM	no	complete	38	Vg	2850	F	Survive	BPG IM, 2.4 mU × 3; Hydrops disappeared in 5 weeks after penicillin first dose (no JHR)

BW: birth weight; CS: cesarean section; d: day; DFU: dead fetus in utero; F: female; FHR: fetal heart rate; GA: gestational age; HD: hydrops; HF: hydrops fetalis; IUT: intrauterine treatment; JHR: Jarisch–Herxheimer reaction; M: male; MCA-PSV: middle cerebral artery peak systolic velocity; ND: normal delivery; NIHF: non-immune hydrops fetalis; Vg: vaginal delivery.

**Table 2:** Summary of the findings.

Continuous Variables	Total Valid Number (N)	Mean	Standard Deviation
Maternal age	30	23.0	5.6
Gestational age at diagnosis of hydrops (week)	29	28.0	3.7
Gestational age at diagnosis of syphilis (week)	23	26.7	4.8
Gestational age at birth (week)	28	32.4	4.8
Birth weight (g)	27	2094	779
**Categorical Variables**	**Total Valid Number (N)**	**Number (n)**	**Percentage**
Parity:	29		
Nulliparous		10	34.5
Parous		19	65.5
MCA-PSV measurement	29	16	55.2
Prenatal treatment	29		
No treatment		10	34.5
Intramuscular BPG		10	34.5
Intravenous PG		9	31.0
Intrauterine blood transfusion	30	5	16.7
Response to therapy	23		
No response		5	21.7
Partial response		5	39.1
Complete response		9	39.1
Route of delivery	27		
Vaginal delivery		13	48.1
Cesarean section		14	51.9
Preterm birth	28	20	71.4
Neonatal sex	25		
Male		13	52.0
Female		12	48.0

## Data Availability

The data of this report are available from the corresponding authors upon request.
